# Supplementary material for: Validation of an AI-based solution for breast cancer risk stratification using routine digital histopathology images
Source: Breast Cancer Res. 2024 Aug 14;26:123. doi: 10.1186/s13058-024-01879-6 (PMC11323658; doi:10.1186/s13058-024-01879-6)
Supplement: Supplementary file 1 — Supplementary Material 1 [file 13058_2024_1879_MOESM1_ESM.pdf]

# Validation of an AI-based solution for breast cancer risk stratification using routine digital histopathology images

Abhinav Sharma[1], Sandy Kang Lövgren[1,5], Kajsa Ledesma Eriksson[1,5], Yinxi Wang[1,5], Stephanie Robertson[2,5], Johan Hartman[2,3,4], Mattias Rantalainen[1,4]

**Corresponding author:** Mattias Rantalainen
Email: mattias.rantalainen@ki.se

**Table of content:**

Table 1. Baseline characteristics of the cohorts from two different hospitals in Sweden.

**Table 1:** Baseline characteristics of the cohorts from two different hospitals in Sweden.

| | CHIME breast KS Solna (N=1670) | SCAN-B (N=1049) | Overall (N=2719) |
|---|---|---|---|
| **Age** | | | |
| Mean (SD) | 61.3 (12.4) | 65.1 (12.4) | 62.8 (12.5) |
| Median [Min, Max] | 63.0 [29.0, 94.0] | 65.0 [30.0, 95.0] | 65.0 [29.0, 95.0] |
| **Tumor_size** | | | |
| <20mm | 1124 (67.3%) | 707 (67.4%) | 1831 (67.3%) |
| >=20mm | 533 (31.9%) | 332 (31.6%) | 865 (31.8%) |
| Missing | 13 (0.8%) | 10 (1.0%) | 23 (0.8%) |
| **Lymph_node** | | | |
| Negative | 1185 (71.0%) | 719 (68.5%) | 1904 (70.0%) |
| Positive | 344 (20.6%) | 298 (28.4%) | 642 (23.6%) |
| Missing | 141 (8.4%) | 32 (3.1%) | 173 (6.4%) |
| **NHG** | | | |
| 1 | 361 (21.6%) | 221 (21.1%) | 582 (21.4%) |
| 2 | 879 (52.6%) | 513 (48.9%) | 1392 (51.2%) |
| 3 | 430 (25.7%) | 315 (30.0%) | 745 (27.4%) |
| **ER_status** | | | |
| Negative | 193 (11.6%) | 109 (10.4%) | 302 (11.1%) |
| Positive | 1470 (88.0%) | 937 (89.3%) | 2407 (88.5%) |
| Missing | 7 (0.4%) | 3 (0.3%) | 10 (0.4%) |
| **HER2_status** | | | |
| Negative | 1462 (87.5%) | 946 (90.2%) | 2408 (88.6%) |
| Positive | 167 (10.0%) | 88 (8.4%) | 255 (9.4%) |
| Missing | 41 (2.5%) | 15 (1.4%) | 56 (2.1%) |
| **PFS_in_years** | | | |
| Mean (SD) | 6.32 (2.52) | 5.34 (2.27) | 5.94 (2.47) |
| Median [Min, Max] | 6.51 [0.153, 10.8] | 5.31 [0.115, 10.2] | 6.15 [0.115, 10.8] |
| **PFS_status** | | | |

| | | | |
|---|---|---|---|
| 0 | 1580 (94.6%) | 1013 (96.6%) | 2593 (95.4%) |
| 1 | 90 (5.4%) | 36 (3.4%) | 126 (4.6%) |

NHG=Nottingham histological grade, ER=oestrogen receptor, HER2=human epidermal growth factor receptor 2, PFS=Progression-free survival.
